# A Systematic Review and Meta-Analysis on the Identification of Predictors Associated With Insomnia or Sleep Disturbance in Post-stroke Patients

**DOI:** 10.7759/cureus.56578

**Published:** 2024-03-20

**Authors:** Pranav Mohandas, Zaid Alomari, FNU Arti, Mohammad Alhneif, Paula Alejandra Ruiz, Alahed K Ahmed, Calvin R Wei, Adil Amin

**Affiliations:** 1 Medicine, Tbilisi State Medical University, Tbilisi, GEO; 2 Internal Medicine, Tbilisi State Medical University, Tbilisi, GEO; 3 Medicine, Ghulam Muhammad Mahar Medical College, Sukkur, PAK; 4 Neuropathology, University of Texas Health Science Center at San Antonio, San Antonio, USA; 5 Public Health, Universidad Nacional de Colombia, Bogota, COL; 6 Faculty of Medicine, Yarmouk University, Irbid, JOR; 7 Research and Development, Shing Huei Group, Taipei, TWN; 8 Cardiology, Pakistan Navy Ship Shifa, Karachi, PAK

**Keywords:** sleep quality, systematic review and meta analysis, predictors, stroke, insomnia

## Abstract

The aim of this study was to identify the factors associated with sleep disturbances in individuals after a stroke. To systematically identify relevant studies, an extensive search strategy was devised. We conducted comprehensive searches in major electronic databases including PubMed, Embase, PsycINFO, and Cochrane Library. The search was limited to articles published in English between January 1, 2011, and February 10, 2024. Pooled effect estimates, such as odds ratio (OR) or mean difference (MD) along with their confidence interval (CIs), were calculated using random-effects models for categorical variables and continuous variables, respectively. A total of nine studies were included in this meta-analysis. The pooled prevalence of insomnia across the included studies was determined to be 40% (95% CI = 30%-49%), with individual study prevalence ranging from 22% to 72%. A pooled analysis showed that gender demonstrated a statistically significant association with sleep disturbance, with females exhibiting a higher likelihood (OR = 1.49, 95% CI = 1.16-1.91, p = 0.002) compared to males. The National Institutes of Health Stroke Scale (NIHSS) score, a measure of stroke severity, was associated with sleep disturbance (MD = 0.86, 95% CI = 0.56-1.17, p = 0.001), indicating that patients with severe strokes may be more prone to sleep disturbances. These findings underscore the importance of comprehensive evaluation and targeted interventions to address sleep-related issues in stroke patients, particularly those with severe neurological impairment.

## Introduction and background

Stroke ranks as the second leading cause of mortality globally, affecting 15 million individuals annually. Among these cases, one-third result in fatalities, another one-third lead to enduring disabilities, while the remaining portion experiences recovery and restoration of normal functions [1]. There are generally considered to be three main types of strokes: ischemic stroke, hemorrhagic stroke, and transient ischemic attack (TIA). This debilitating ailment imposes both physical and emotional strains on stroke survivors and their caregivers, manifesting in physical impairments, functional reliance, and emotional and sleep-related disorders [2]. Disturbances in sleep patterns post-stroke are frequently reported yet underexplored phenomena [3], which are recognized as both precursors and outcomes of stroke [4-5]. These disruptions are believed to hinder the functional recuperation of daily activities, mood regulation, and fatigue levels [6], with stroke survivors reporting alterations in sleep duration and patterns [7].

Despite the correlation between sleep disturbances and stroke, the prevalence of such disturbances among stroke patients remains poorly defined. Existing studies are often constrained by small sample sizes and encompass diverse stroke characteristics and populations, resulting in prevalence estimates ranging from 25% to 80% [8-9]. Meta-analyses have suggested a prevalence of post-stroke insomnia ranging from 29% to 54%, disregarding those experiencing post-stroke hypersomnia [10]. Insomnia has been linked to diminished health-related quality of life (HRQoL) across various populations, including the elderly and individuals with Parkinson’s disease [11-14].

Given the significant impact of insomnia on quality of life during stroke recovery, promptly identifying and addressing sleep disturbances in this demographic by identifying high-risk individuals through early risk factor assessment. However, despite its importance, there is a dearth of studies investigating the specific risk factors contributing to sleep disorders in stroke patients. Therefore, we have initiated a systematic review and meta-analysis of existing literature to elucidate the factors associated with insomnia or sleep disturbances in individuals following a stroke.

## Review

Search strategy

To systematically identify relevant studies, we devised an extensive search strategy. We conducted comprehensive searches in major electronic databases, such as PubMed, Embase, PsycINFO, and Cochrane Library. The search strategy involved combining Medical Subject Headings (MeSH) terms and keywords related to stroke ("stroke" OR "cerebrovascular accident" OR “ischemic stroke” OR “hemorrhagic stroke”) and sleep disturbance ("sleep disorders" OR "insomnia") (search strategy attached in Appendix 1). The search was limited to articles published in English between January 1, 2011, and February 10, 2024. The selected databases were searched systematically, and efforts were made to minimize the risk of missing relevant studies by employing a combination of controlled vocabulary and free-text terms. The search was performed by two authors (MA and AK) independently, and any disagreement between the two authors was resolved through discussion or consultancy with the principal investigator (PM).

Study selection

Inclusion criteria were defined to ensure the relevance of the selected studies to our research question. We included studies based on the PECOS (population, exposure, comparison, outcome, study design) criteria. The population comprised individuals with stroke. The exposure and comparison group included risk factors associated with insomnia or sleep disturbance. The comparison group included patients without insomnia or sleep disturbance. Outcomes included insomnia or sleep disturbance. Studies involving pediatric populations or non-stroke patients were excluded. The initial screening involved assessing titles and abstracts for relevance, followed by a full-text review of potentially eligible studies. The screening process was conducted independently by two reviewers (PR and AA), with any discrepancies resolved through discussion or consultation with a third reviewer (PM).

Data extraction

A standardized data extraction form was developed to systematically capture relevant information from each included study. The data extraction form included variables such as study characteristics (author, year of publication), participant demographics (age, sex), stroke characteristics (subtype, severity), sleep disorder diagnosis criteria, and identified risk factors. Two independent reviewers (ZA and CW) performed data extraction from selected studies, and any discrepancies were resolved through consensus or consultation with a third reviewer (PM). Efforts were made to ensure accuracy and completeness in capturing relevant data from each study.

Quality assessment

The quality of the included studies was assessed using the Newcastle-Ottawa Scale (NOS) for observational studies [15]. The NOS evaluates the methodological quality of studies based on three domains: selection of participants, comparability of study groups, and assessment of outcome or exposure. Each study was independently assessed by two reviewers (AA and ZA), with discrepancies resolved through discussion. Studies were assigned a score based on predefined criteria, with higher scores indicating higher methodological quality. Studies with low quality scores were not excluded from the review but were considered in the interpretation of results and discussion of potential biases.

Data synthesis and analysis

To perform the meta-analysis, we used RevMan (version 5.4; The Cochrane Collaboration, Copenhagen, Denmark) [16]. Meta-analysis was performed to synthesize data from included studies and quantify the association between identified risk factors and sleep disorders in stroke patients. Pooled effect estimates, such as odds ratio (OR) or mean difference (MD) along with their confidence interval (CI), were calculated using random-effects models for categorical variables and continuous variables, respectively. We used the random-effect model to deal with heterogeneity among the study results potentially due to varying sample sizes, study designs, study populations, and tools to measure sleep disturbance. The I-squared statistic was used to assess heterogeneity, with values greater than 50% considered indicative of substantial heterogeneity.

Results

**Figure 1:**
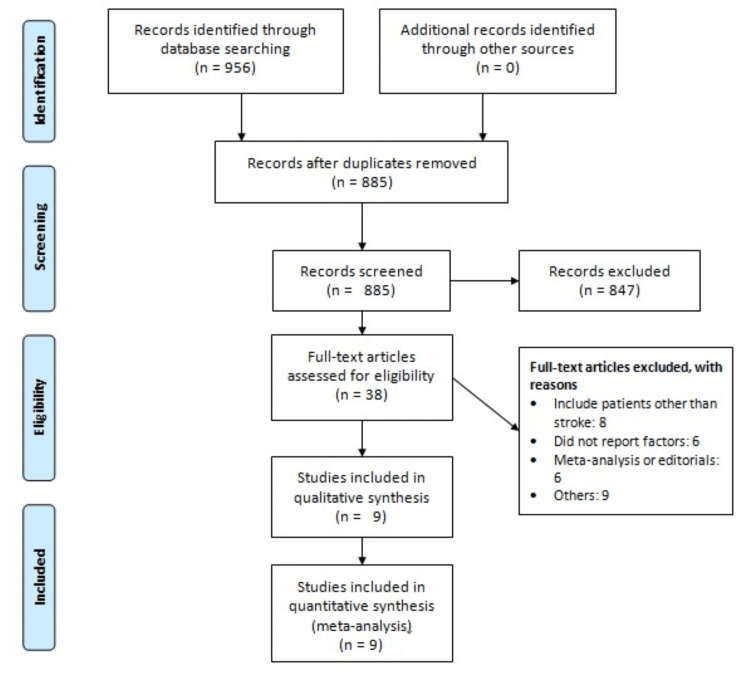
PRISMA flowchart showing the study selection process

Figure 1 illustrates the flowchart detailing the study selection process. Initially, 956 articles were identified. Following the removal of duplicates, their abstracts and titles underwent screening for inclusion. Subsequently, the full texts of 38 articles were deemed eligible for review. Ultimately, nine studies met the criteria for inclusion in this meta-analysis. Table 1 shows the characteristics of the included studies. These selected studies were published between 2011 and 2023, predominantly originating from China (n = 4). The pooled prevalence of insomnia across the included studies was determined to be 40% (95% CI = 30%-49%), with individual study prevalence ranging from 22% to 72%. For a comprehensive overview of the characteristics of the included studies, refer to Table 2. Additionally, Table 3 provides a quality assessment of all of the included studies.

**Table 1:** Characteristics of included studies PSQI: Pittsburgh Sleep Quality Index; NS: Not specified; KSQ: Karolinska Sleep Questionnaire; DSM-V: Diagnostic and Statistical Manual of Mental Disorders, Fifth Edition; DSM-IV: Diagnostic and Statistical Manual of Mental Disorders, Fourth Edition

Author	Year	Study Design	Insomnia Measure	Region
Al Battat et al. [17]	2023	Cross-sectional	PSQI	Jordan
Chen et al. [18]	2011	Cross-sectional	NS	China
Glozier et al. [19]	2017	Prospective	KSQ	Australia
Kalmbach et al. [20]	2016	Cross-sectional	DSM-V	United States
Li et al. [21]	2018	Retrospective	DSM-IV	China
Moon et al. [22]	2018	Retrospective	DSM-IV	Korea
Tang et al. [23]	2015	Cross-sectional	NS	China
Tayade et al. [24]	2023	Cross-sectional	PSQI	Switzerland
Xiao et al. [25]	2020	Retrospective	PSQI	China

**Table 2:** Summary of findings of included studies NIHSS: National Institutes of Health Stroke Scale

Study ID	Sample Size	Insomnia	Variables Assessed
Al Battat et al. [17]	94	56	Age, gender, BMI, marital status, diabetes, hypertension, smoker
Chen et al. [18]	490	168	Age, gender, marital status, alcohol user, smoker, hypertension, diabetes, NIHSS
Glozier et al. [19]	368	124	Gender, marital status, alcohol user, smoker
Kalmbach et al. [20]	3,551	806	Age, hypertension
Li et al. [21]	1,062	489	Gender, marital status, smoker, diabetes, NIHSS
Moon et al. [22]	140	35	Age, gender,
Tang et al. [23]	336	149	Age, gender, hypertension, diabetes, NIHSS
Tayade et al. [24]	103	74	Age, gender, alcohol user, smoker, hypertension, diabetes, BMI
Xiao et al. [25]	327	76	Age, gender, marital status, alcohol user, smoker, hypertension, diabetes, NIHSS

**Table 3:** Quality assessment of included studies

Author	Selection	Comparison	Assessment	Overall
Al Battat et al. [17]	4	2	3	Good
Chen et al. [18]	2	2	2	Fair
Glozier et al. [19]	4	1	3	Good
Kalmbach et al. [20]	3	2	2	Good
Li et al. [21]	4	2	2	Good
Moon et al. [22]	3	2	2	Good
Tang et al. [23]	3	2	3	Good
Tayade et al. [24]	4	1	2	Good
Xiao et al. [25]	3	2	3	Good

Factors associated with insomnia in patients with stroke

**Figure 2:**
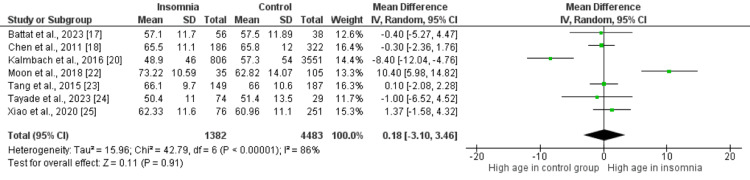
Comparison of mean age between insomnia and control groups Sources: References [17,18,20,22-25]

**Figure 3:**
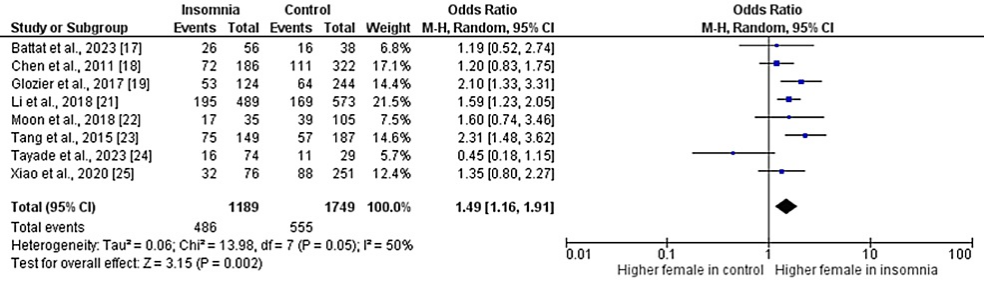
Comparison of the number of females between insomnia and control groups Sources: References [17-19,21-25]

**Figure 4:**
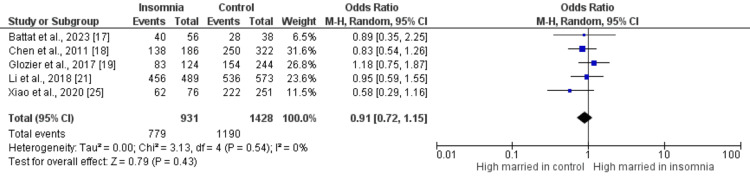
Comparison of the number of married individuals between insomnia and control groups Sources: References [17-19,21,25]

**Figure 5:**
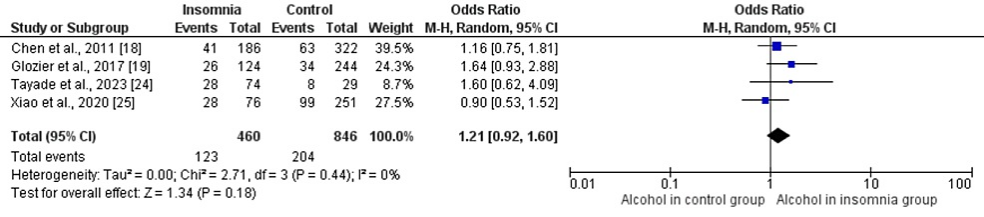
Comparison of the number of alcohol users between insomnia and control groups Sources: References [18,19,24,25]

**Figure 6:**
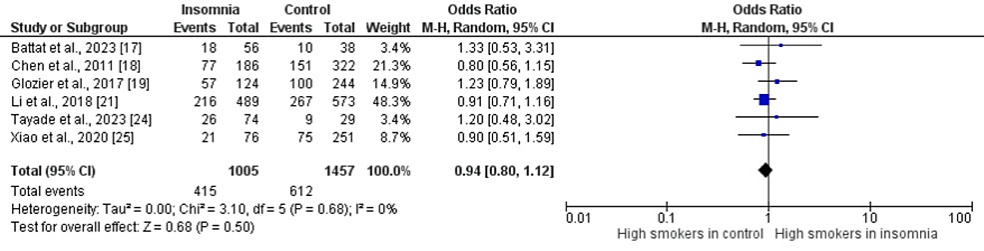
Comparison of the number of smokers between insomnia and control groups Sources: References [17-19,21,24,25]

**Figure 7:**
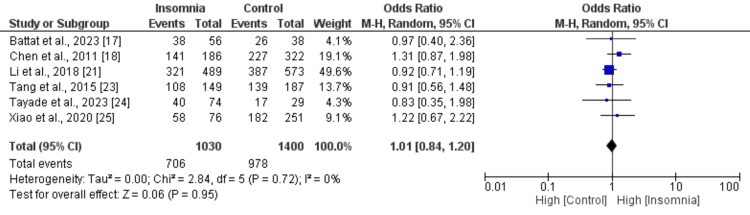
Comparison of the number of hypertensive patients between insomnia and control groups Sources: References [17,18,21,23-25]

**Figure 8:**
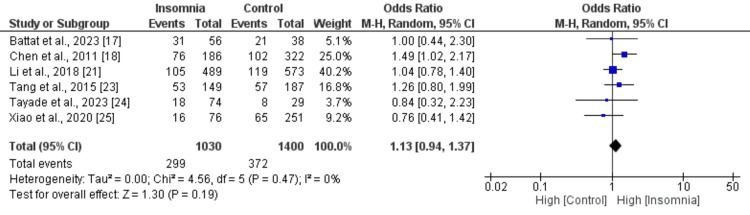
Comparison of diabetes between insomnia and control groups Sources: References [17,18,21,23-25]

**Figure 9:**
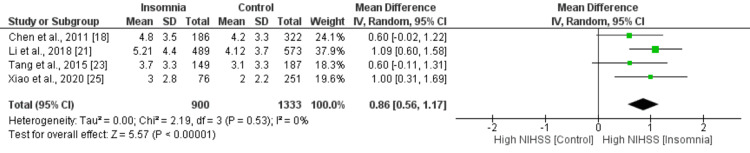
Comparison of the NIHSS scores between insomnia and control groups Sources: References [18,21,23,25]

**Figure 10:**
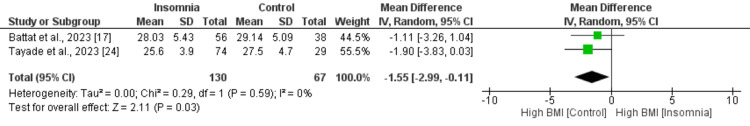
Comparison of BMI scores between insomnia and control groups Sources: References [17,24]

Figures 2-10 show the results of factors associated with sleep disturbance in stroke patients. Among the factors analyzed, gender demonstrated a statistically significant association with sleep disturbance, with females exhibiting a higher likelihood (OR = 1.49, 95% CI = 1.16-1.91, p = 0.002) compared to males. Notably, age, marital status (specifically being married), alcohol use, smoking status, hypertension, and diabetes did not demonstrate significant associations with sleep disturbance in this patient population.

**Figure 11:**
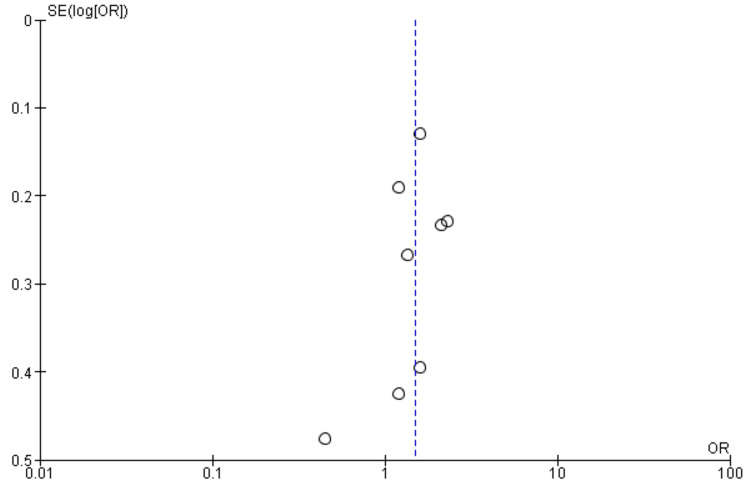
Funnel plot showing publication bias for variable gender

**Figure 12:**
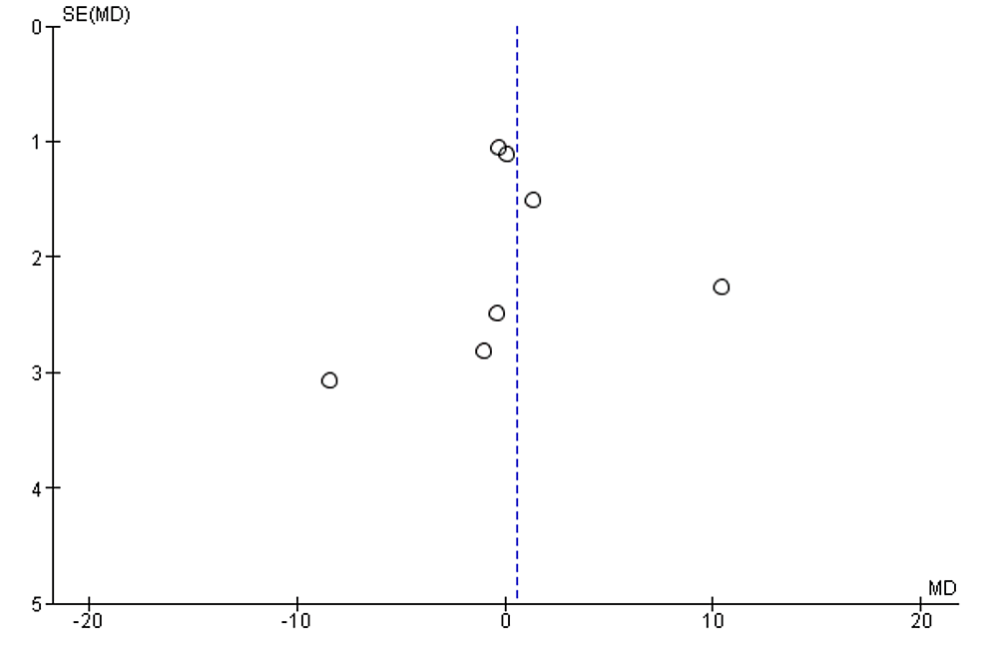
Funnel plot showing publication bias for variable age

**Figure 13:**
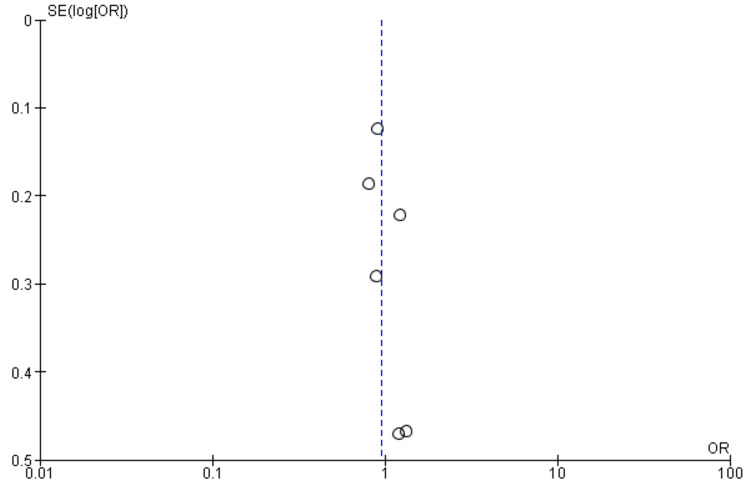
Funnel plot showing publication bias for variable smoking

**Figure 14:**
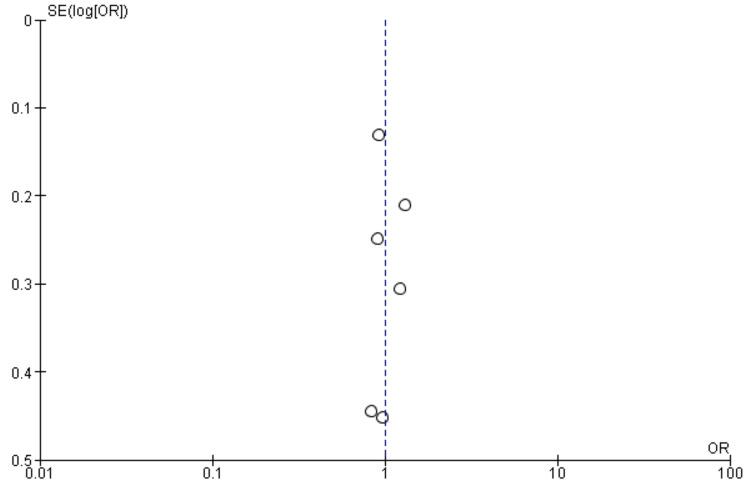
Funnel plot showing publication bias for variable hypertension

**Figure 15:**
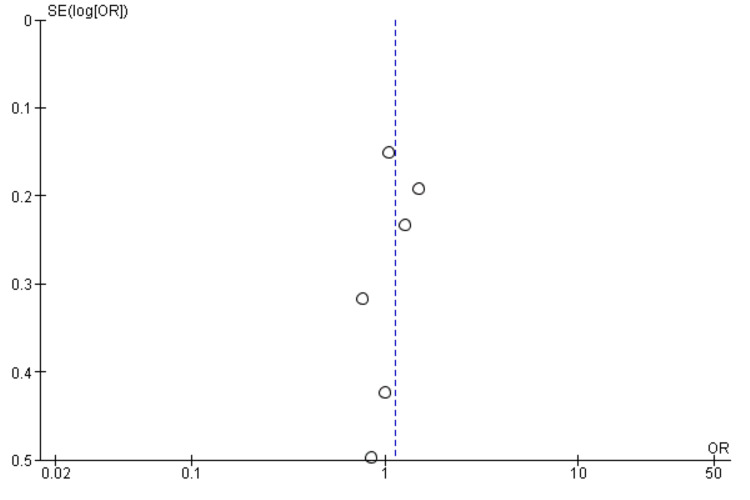
Funnel plot showing publication bias for variable diabetes

Interestingly, the National Institutes of Health Stroke Scale (NIHSS) score, a measure of stroke severity, was associated with sleep disturbance (MD = 0.86, 95% CI = 0.56-1.17, p = 0.001). This indicates that patients with severe strokes may be more prone to sleep disturbances. Furthermore, body mass index (BMI) exhibited a statistically significant negative association with sleep disturbance (MD = -1.55, 95% CI = -2.99 to -0.11, p = 0.03), suggesting that lower BMI may be associated with a higher likelihood of sleep disturbances post-stroke. Figures 11-15 present the funnel plot demonstrating the publication bias for variables that were assessed by more than five studies (gender, age, smoking, hypertension, and diabetes).

To the best of our knowledge, this was the first systematic review and meta-analysis determining the factors associated with sleep disorders in patients with stroke. The pooled prevalence of sleep disturbance in our meta-analysis was 40% (95% CI = 30%-49%), while the prevalence of sleep disturbance reported in individual studies included in this meta-analysis ranged from 22% to 72%. The findings of this meta-analysis showed that the risk of sleep disorder was significantly higher in females. Furthermore, stroke severity and BMI also emerged as significant factors as individuals with more severe strokes and low BMI were more prone to sleep disturbances.

In line with prior research [26], our findings demonstrate a notably higher prevalence of sleep disorders among stroke survivors compared to the general population [27]. However, the actual prevalence of sleep disorders may have been inaccurately estimated because some studies included in our analysis failed to exclude participants with preexisting sleep disorders before experiencing a stroke. The relationships between sleep-disordered breathing (SDB) and stroke are multifaceted [28,29]. Mounting evidence suggests that stroke can instigate or exacerbate preexisting sleep disorders through various mechanisms, including impairment of brain stem respiratory drive centers (e.g., medullary lesions) [30], dysfunction of the hypoglossal nerve [29], disrupted coordination of the upper airway, diminished voluntary chest movement on the affected side [31], and prolonged periods in the supine position [32]. These factors may also play pivotal roles in the onset of SDB following a stroke or TIA. To address sleep disorders in stroke patients, individuals should minimize exposure to noise and light, opting for quiet environments during nighttime hours. One recommended treatment is cognitive-behavioral therapy (CBT), which has shown effectiveness in managing chronic insomnia in stroke patients requiring long-term care. However, the sustained physical and mental benefits of CBT post-stroke remain unclear and warrant further investigation with larger sample sizes in future studies [2,33].

Our meta-analysis reveals a notable discrepancy in the prevalence of sleep disorders between females and males within the stroke patient population. Our findings suggest a higher likelihood of sleep disorders among females compared to males. This observation aligns with established research in the general population, where being female has been independently associated with a higher prevalence of insomnia [34,35]. However, it is important to note that the relationship between female sex and self-reported insomnia specifically among stroke patients has not been previously documented in the literature [36,37]. This disparity in findings between our study and prior research involving insomnia patients could potentially be attributed to methodological disparities. Previous studies might have focused on narrower aspects of sleep disorders or utilized different methodologies for assessing sleep disturbances among stroke patients. Additionally, our meta-analysis adopts a broader focus by encompassing various types of sleep disorders, whereas prior studies might have concentrated solely on insomnia. These methodological differences could contribute to variations in observed associations between female sex and sleep disturbances among stroke patients. Further research employing standardized methodologies and comprehensive assessments of sleep disorders in stroke populations is warranted to elucidate this relationship more definitively.

The present study's discovery, indicating a notable increase in the risk of sleep disorder among patients with higher NIHSS scores, is consistent with earlier research findings. Numerous studies have established a link between stroke severity, as determined by NIHSS scores, and the frequency and intensity of sleep disruptions following a stroke [19]. Elevated NIHSS scores are indicative of greater neurological damage, which may disrupt mechanisms regulating sleep and raise the probability of developing sleep disorders [11]. The physiological and structural changes that occur in the brain because of stroke can disrupt the normal sleep-wake cycle. Additionally, the emotional and psychological impact of a stroke can lead to increased levels of stress, anxiety, and depression, all of which can contribute to insomnia [26]. Additionally, the physical impairments resulting from a stroke, such as pain, discomfort, and mobility limitations, can make it difficult for individuals to find a comfortable sleep position [38]. These results emphasize the significance of evaluating stroke severity when addressing sleep disturbances and underscore the necessity for tailored interventions to tackle sleep-related concerns among stroke patients exhibiting more pronounced neurological impairment.

The study's findings hold crucial clinical and research implications. Highlighting a significantly higher prevalence of sleep disorders in stroke patients, particularly among females, underscores the importance of targeted screening and intervention strategies. Understanding associations with stroke severity and BMI offers avenues for personalized treatment approaches. Further research is warranted to explore the nuanced relationships between sleep disorders and stroke outcomes, guiding the development of tailored interventions to enhance patient care and quality of life.

Study limitations

The current meta-analysis comes with certain limitations. First, none of the studies included in this analysis directly compared medications between the two groups. Various drugs commonly used to manage stroke, or its associated conditions may influence sleep patterns. For instance, stroke patients with hypertension often take medications such as beta-blockers, clonidine, or diuretics, which can disrupt rapid eye movement (REM) sleep, induce insomnia, and result in early morning awakenings, nightmares, or painful calf cramps during sleep. Similarly, stroke patients with psychiatric symptoms may be prescribed selective serotonin reuptake inhibitors (SSRIs), such as sertraline or paroxetine, which have been shown to decrease REM sleep and increase daytime fatigue. Further research is needed to investigate the specific effects of different medications on insomnia among stroke patients. Secondly, because of the limited number of studies included in our analysis for estimating the factors associated with sleep disorders in stroke patients, it is important to interpret our findings cautiously. Additional investigations are warranted to enhance our understanding in this area. Moreover, data on the prevalence of sleep disorders among African and Eastern populations, including Russian and South Asian populations, were scarce. This highlights the need for further research to better comprehend the prevalence and characteristics of sleep disorders in these demographic groups.

## Conclusions

In conclusion, our meta-analysis revealed significant associations between various factors and sleep disturbances in stroke patients. While gender, stroke severity as measured by NIHSS scores, and BMI emerged as significant predictors of sleep disturbances, other factors such as age, marital status, alcohol use, smoking status, hypertension, and diabetes did not exhibit significant associations. These findings underscore the importance of comprehensive evaluation and targeted interventions to address sleep-related issues in stroke patients, particularly those with severe neurological impairment. However, our analysis is subject to limitations, including the lack of comparative studies on the effects of different medications on insomnia in stroke patients and the scarcity of data on sleep disorders prevalence in certain populations. Further research is warranted to better understand these complexities and improve management strategies for sleep disturbances in stroke patients.

## Appendices

Appendix 1

Search Strategy for PubMed

("Stroke"[Mesh] OR "Cerebrovascular Accidents"[Mesh]) AND ("Sleep Disorders"[Mesh]) AND (predict* OR determinant* OR "risk factors"[Mesh]) AND (("stroke patient*"[Text Word] OR "post-stroke"[Text Word])).
